# Molybdenum distributions and variability in drinking water from England and Wales

**DOI:** 10.1007/s10661-014-3863-x

**Published:** 2014-07-11

**Authors:** P. L. Smedley, D. M. Cooper, D. J. Lapworth

**Affiliations:** 1 British Geological Survey, Keyworth, Nottingham, NG12 5GG UK; 2 Centre for Ecology and Hydrology, Bangor, Gwynedd LL57 2UW UK; 3 British Geological Survey, Wallingford, Oxfordshire OX10 8BB UK

**Keywords:** Trace metals, Groundwater, Surface water, Health, Monitoring, WHO

## Abstract

An investigation has been carried out of molybdenum in drinking water from a selection of public supply sources and domestic taps across England and Wales. This was to assess concentrations in relation to the World Health Organization (WHO) health-based value for Mo in drinking water of 70 μg/l and the decision to remove the element from the list of formal guideline values. Samples of treated drinking water from 12 water supply works were monitored up to four times over an 18-month period, and 24 domestic taps were sampled from three of their supply areas. Significant (*p* < 0.05) differences were apparent in Mo concentration between sources. Highest concentrations were derived from groundwater from a sulphide-mineralised catchment, although concentrations were only 1.5 μg/l. Temporal variability within sites was small, and no seasonal effects (*p* > 0.05) were detected. Tap water samples collected from three towns (North Wales, the English Midlands, and South East England) supplied uniquely by upland reservoir water, river water, and Chalk groundwater, respectively, also showed a remarkable uniformity in Mo concentrations at each location. Within each, the variability was very small between houses (old and new), between pre-flush and post-flush samples, and between the tap water and respective source water samples. The results indicate that water distribution pipework has a negligible effect on supplied tap water Mo concentrations. The findings contrast with those for Cu, Zn, Ni, Pb, and Cd, which showed significant differences (*p* < 0.05) in concentrations between pre-flush and post-flush tap water samples. In two pre-flush samples, concentrations of Ni or Pb were above drinking water limits, although in all cases, post-flush waters were compliant. The high concentrations, most likely derived from metal pipework in the domestic distribution system, accumulated during overnight stagnation. The concentrations of Mo observed in British drinking water, in all cases less than 2 μg/l, were more than an order of magnitude below the WHO health-based value and suggest that Mo is unlikely to pose a significant health or water supply problem in England and Wales.

## Introduction

Drinking water in the UK is monitored regularly in compliance with the requirements of the national and European drinking water legislation. However, monitoring effort is naturally concentrated on chemical constituents covered by the legislation. For those that are not covered, monitoring and consequent availability of data are comparatively sparse. The non-regulated trace elements include molybdenum (Mo), for which few data exist in British drinking water supplies despite uncertainties over the element’s potential health impacts.

Molybdenum is an essential trace element for human health, but, as with many elements, high doses can be detrimental. Adults have an estimated daily requirement for Mo of 75–250 μg (National Academy of Sciences 1989). The element is important to the functioning of the enzymes xanthine dehydrogenase, sulphite oxidase, and aldehyde oxidase, which play key roles in human metabolism (Expert Group on Vitamins and Minerals 2003; Momcilovic 1999; WHO 2011a, b). It also has potential benefits for patients with asthma and sulphite sensitivity. However, chronic occupational exposure has been linked to a number of ailments including fatigue, lack of appetite, anorexia, joint pain, and tremor. Exposure may also give rise to Mo-induced copper deficiency and pneumoconiosis (Expert Group on Vitamins and Minerals 2003; WHO 2011b). The chemical state of Mo, route of exposure, and dietary doses of copper and sulphur all likely have an impact on its toxicity. Despite the above observations, recognised cases of Mo toxicity in humans are rare.

The *1993 WHO guidelines for drinking water quality* (second edition) introduced a health-based guideline value for Mo in drinking water of 70 μg/l. The 2011 fourth edition of the guidelines continues to advise a health-based value of 70 μg/l, consistent with the toxicological evidence and the essential daily requirement for molybdenum (WHO 2011a, b). However, the World Health Organization (WHO) considers recommending a formal guideline value no longer necessary on the grounds that such concentrations are rarely found in drinking water.

A recent investigation of Mo distributions in surface water and groundwater across Great Britain has found that concentrations are usually low and typically less than 2 μg/l (Smedley et al. 2014). This would be consistent with the WHO conclusion that concentrations are rarely high enough in drinking water to warrant the retention of a formal guideline value. However, the study also found that concentrations could be higher, potentially approaching 70 μg/l, in water sources impacted by sulphide mineralisation and/or industrial contamination. This study investigates the spatial and temporal variability in Mo concentrations in a sample of public supply drinking waters from England and Wales including treated water from supply works and from consumers’ taps. The study aims to assess whether concentrations in tap water are consistent with those observed in surface water and groundwater sources and whether contact with metallic pipework in the distribution system has an impact on Mo concentrations as identified by comparison of results at each end of the supply network. The study also assesses the implications for water supply in the context of the WHO guidelines for drinking water quality.

## Drinking water survey design

### Treated public supply water survey

Available data for surface water and groundwater in Britain, collated from available Natural Environment Research Council (NERC) databases, including the NERC Land-Ocean Interaction Study (Neal et al. 2003; Neal and Robson 2000; Wilkinson et al. 1997), the UK Environmental Change Network (ECN) (http://www.ecn.ac.uk; Lane 1997), and the British Geological Survey (BGS) stream water (Johnson et al. 2005) and groundwater data, were evaluated by Smedley et al. (2014). The available data (Table 1) suggest that concentrations in surface water and groundwater are usually low, typically <2 μg/l, although occasional high values are observed. These are most often derived from low-order streams in sulphide-mineralised areas, in proximity to mine wastes and affected by mine drainage, under low-flow conditions in some river waters from industrial areas (e.g. River Aire, Yorkshire; Table 1) and in some anaerobic groundwaters. However, for each water type (streams, rivers, lakes, and groundwater), the proportion of analyses with concentrations  > 70 μg/l was extremely small (Smedley et al. 2014).

**Table 1:** Ranges and median values for molybdenum in British surface water and groundwater

Water type	Range (μg/l)	Median (μg/l)	Number (*n*)	Reference
Low-order streams, England and Wales	<0.05–230	0.57	11,562	BGS G-BASE data (Johnson et al. 2005)
Low-order streams, Northern Ireland	<0.02–27.7	0.16	5,892	GSNI data (A. Donald, personal communication)
Upland streams (baseflow), Wales	0–14.7	0.20	67	Neal et al. (1998)
Upland stream (storm flow), Wales	0–11.2	0.36	67	
River Tweed (Teviot)	0–4.18	0.25	119	Neal and Robson (2000)
River Wear	0.20–10.3	0.80	55	
River Swale (Catterick)	0–5.00	0.29	172	
River Nidd	0–4.32	0.84	184	
River Ure	0–3.0	0.37	180	
River Ouse (Acaster)	0–4.47	0.64	144	
River Derwent	0–26	0.57	173	
River Wharfe	0–4.92	0.56	192	
River Aire	0.32–70.3	20.7	196	
River Calder	0.57–19.7	3.40	176	
River Don	0.70–20.1	8.34	180	
River Trent	1.75–9.80	5.27	153	
River Great Ouse	1.1–40.2	2.46	58	
River Thames (Oxfordshire)	0.5–10.0	2.16	108	
Esthwaite Water (lake)	0.069–0.162	0.099	32	ECN data (P. Rowland, personal communication)
Windermere (lake, north bank)	0.048–0.150	0.086	32	
Windermere (lake, south bank)	0.050–0.157	0.094	32	
British groundwater	<0.02–89.2	0.20	1,735	Smedley et al. (2014)

The main purpose of the current survey of public water supply sources was to identify the risk of exceedance of the WHO health-based value of 70 μg/l Mo. Evidence from the available surface water and groundwater data suggests that, at a national scale, the probability of exceedance in water from any source is small. Estimation of this proportion on a national scale, using simple random sampling, and scoring each site according to pass or fail would require a large sample to determine this small proportion with reasonable accuracy. Formal stratification would also require a relatively large sample. Given available resources, a purposive sampling approach was adopted, focusing instead on those regions where the risk of exceedance was believed to be highest on the basis of pre-existing source water data.

Of the raw groundwaters investigated, the most prominent aquifers at risk appeared to be parts of the Cretaceous Greensand of Lincolnshire, Chalk of East Anglia, Carboniferous of Northern England and Derbyshire, and the Triassic Sandstone of the West Midlands. As a result, public supplies abstracting groundwater from these areas were targeted for monitoring.

It was considered highly unlikely that upland reservoirs have Mo concentrations above the WHO health-based value. However, apart from parts of North West England (Cumbria, ECN data, Table 1), few data existed for upland areas before the survey was carried out. Data from the BGS G-BASE database (Johnson et al. 2005) suggest that, although uncommon, relatively high Mo concentrations exist in low-order streams draining parts of North Wales and the Peak District of Derbyshire. Public supply sources from these areas were therefore also selected as upland reservoir monitoring sites.

River sources flowing through industrial areas, particularly, former mining areas, were also targeted for monitoring. For rivers, it was considered important that sampling included low-flow as well as high-flow samples, in view of the evidence (Neal et al. 2003; Neal and Robson 2000; Wilkinson et al. 1997) that for many rivers, concentrations of many solutes are higher under low-flow conditions (notwithstanding the fact that water abstracted at low flow from rivers and stored in reservoirs may have undergone some dilution through mixing of waters of varying residence times).

### Domestic tap water survey

The purpose of the tap water survey was to determine whether there is any effect of pipework on Mo concentrations in drinking water. This is most easily achieved by measuring the difference in concentrations between water leaving a treatment works and water delivered to household taps. Molybdenum might either be lost or gained in the system, and this might depend on both the nature of the pipework and the chemical composition of the source water. It may also depend on the residence time of water in the pipework.

In the absence of any knowledge of possible changes in the supply line, it was considered sensible to investigate a limited number of areas with known supply sources, with several taps in each area. Sampling design therefore incorporated taps supplied from three of the surveyed supply areas, one from each of the main water source types (groundwater, river, and upland reservoir). This would be most likely to encompass samples with variable major ion chemistry (e.g. soft versus hard water). It was also desirable to select supply sources with variable Mo concentrations. Taps were selected from a mixture of households with old and new plumbing, and sampling included first morning draw from the mains and after flushing to clear pipework within the building. Some near-replication was attempted by sampling within the same street (assuming the same mains source and similar plumbing). Actual site selection depended heavily on obtaining permission and access to sample from householders.

## Sampling and analytical methodology

### Public supply survey

**Fig. 1:**
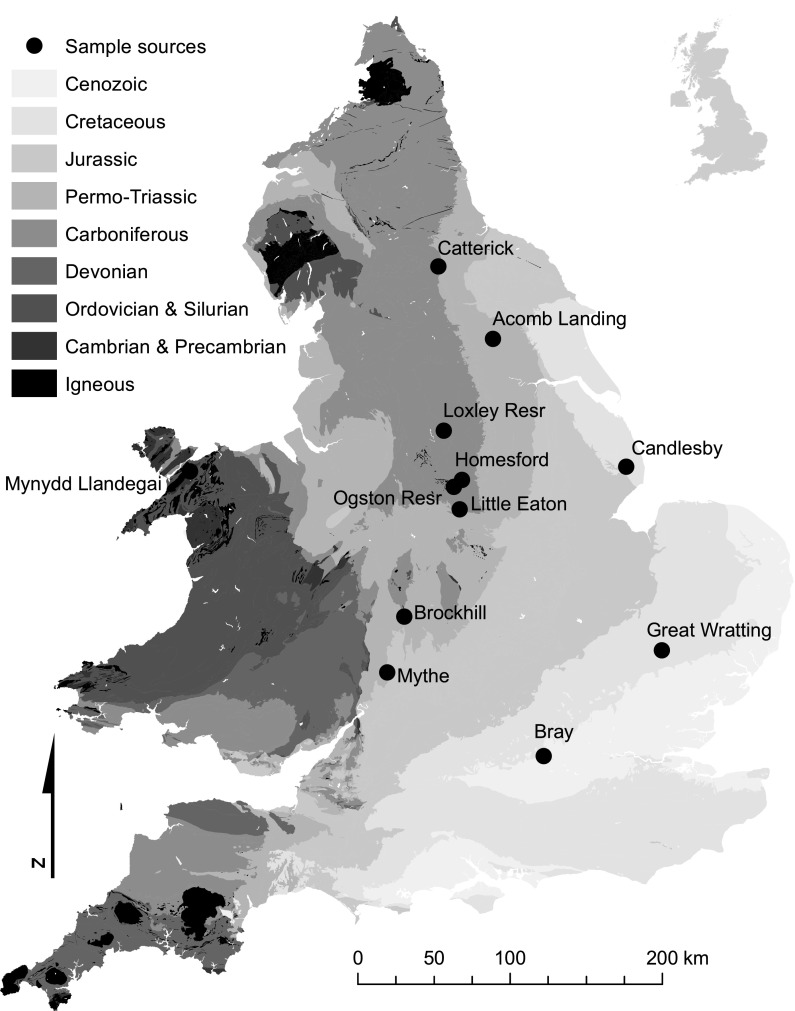
Simplified geology of England and Wales showing sampling locations of the 12 public supply drinking water sources

Public supply water treatment works selected for sampling were spread across England and Wales (Fig. 1). There were 12 sites visited, 11 of which were sampled four times. Sampling dates were March–May 2007, July 2007, November–December 2007, and March 2008. Of the 12 sites, five were from groundwater sources (four boreholes and one mine ‘sough’ or drainage tunnel), four from river sources, and three from upland reservoirs. At the time of sampling, supply water from these sources underwent treatment which varied between sites, but included granular activated carbon, lime/ferrous sulphate coagulation, and phosphate dosing at some sites and chlorination at all.

Samples of the treated water supply were taken from the treatment works after the tap had been allowed to flush. Samples were collected in factory-new acid-washed HDPE bottles, pre-dosed with Aristar™ nitric acid, to a final volume of 1 %. Duplicate samples were taken at three of the sites, and two field blanks (separate sampling rounds) were taken through the same procedure as the samples. Samples were refrigerated before analysis. During the sampling, on-site measurements were also made of electrical conductivity, alkalinity (as HCO_3_, by titration with H_2_SO_4_), and pH.

Collected water samples were pretreated by heating overnight at 80 °C to dissolve any particulate matter and trace elements adsorbed to bottle walls. Bottles were allowed to cool and aliquots were then decanted into acid-washed and rinsed LDPE bottles ready for analysis. In addition, a 10 μg/l standard solution was prepared with each sample batch and analysed along with the batches. Heated (80 °C) and unheated aliquots of this solution were analysed for comparison.

### Domestic tap water survey

Three towns/suburbs were selected for the tap water survey. These were Bangor (Gwynedd), supplied by Mynydd Llandegai treatment works, Mickleover (Derbyshire), supplied by Little Eaton treatment works, and Haverhill (Suffolk), supplied by Great Wratting treatment works. The selection was made on the basis that supply areas were clearly identifiable and encompassed sources from upland reservoir, river water, and groundwater.

Samples were taken from eight domestic properties in each of the three areas (i.e. 24 domestic taps). Sampling was carried out during November–December 2007, coincident with the third monitoring round of the public supply sources. In each source area, four taps were from relatively modern houses (post-1990) and four from older houses (pre-1960). Both pre-flush and post-flush samples were taken from each tap. The pre-flush sample was collected first thing in the morning in order to assess the chemical composition of water that had been in the pipes overnight. The post-flush sample was taken after the tap had been allowed to run to waste for 2–3 min. In each case, samples were from the normal source of drinking water in the household (usually the kitchen tap).

Pre-flush samples were collected in 1 l acid-washed HDPE bottles, and post-flush in 125 ml HDPE. Samples were acidified (1 % v/v HNO_3_) in the laboratory as soon as possible after collection. The subsequent protocol was identical to that for the public supply samples. Laboratory blanks and standards were processed along with the tap water samples.

## Chemical analysis

All samples were analysed for Mo and other trace elements using a Perkin-Elmer DRCII ICP-MS instrument. The detection limit for Mo in all sample batches was 0.03 μg/l (4*σ* on the blank) except for two samples which were 0.06 μg/l due to sample dilution. During the course of the analysis, eight measurements of CRM SLRS-4 gave a mean value for Mo of 0.19 μg/l with a standard deviation of 0.01 μg/l [certified value 0.21 ± 0.02 (2*σ*) μg/l]. Molybdenum concentrations in laboratory blanks were, in most cases, <0.03 μg/l and, in all cases, <0.06 μg/l. The two field blanks also had Mo concentrations < 0.03 μg/l. No blank correction was applied to the data.

Prepared 10 μg/l standard solutions (unheated) gave a mean value of 9.44 μg/l (1*σ* 0.24 μg/l); four standard solutions pre-heated at 80 °C gave a mean value of 9.53 μg/l (1*σ* 0.13 μg/l). Results for the three duplicate samples showed variations of <5 % between Mo concentrations.

## Results

### Treated public supply sources

Selected chemical data for the four public supply sampling rounds are given in Table 2. Waters from the survey sites had mostly near-neutral pH values (most acidic Brockhill, pH range 6.5–6.7; most alkaline Little Eaton, pH 7.0–8.3). Electrical conductivity (EC) measurements show that the least mineralised waters were from the upland reservoir works at Mynydd Llandegai (71–97 μS/cm), while the most mineralised were from the Chalk groundwater works at Great Wratting (929–1,120 μS/cm).

**Table 2:** Monitoring data for Mo and parameters measured on-site in treated waters from the 12 public supply sites

Locality	Source type	Sample date	Round	pH	EC (μS/cm)	HCO_3_ (mg/l)	Mo (μg/l)
Acomb Landing	River	01-May-07	1	7.08	446	117	0.281
Acomb Landing	River	18-Jul-07	2	7.09	484	89.0	0.364
Acomb Landing	River	27-Nov-07	3	6.87	498	101	0.203
Acomb Landing	River	12-Mar-08	4	7.10	330	61.6	0.126
Acomb Landing	River	12-Mar-08	4				0.132
Bray	River	03-Apr-07	1	7.38	747	154	0.587
Bray	River	19-Jul-07	2	7.80	625	241	1.05
Bray	River	29-Nov-07	3	7.34	699	223	0.639
Bray	River	14-Mar-08	4	7.31	690	219	0.644
Brockhill	Groundwater	04-Apr-07	1	6.51	474	80.5	<0.03
Brockhill	Groundwater	16-Jul-07	2	6.60	460	84.1	<0.03
Brockhill	Groundwater	16-Jul-07	2	6.60	460	84.1	<0.03
Brockhill	Groundwater	26-Nov-07	3	6.71	453	135	<0.03
Brockhill	Groundwater	10-Mar-08	4	6.52	435	118	<0.03
Candlesby	Groundwater	26-Mar-07	1	7.46	708	215	0.849
Candlesby	Groundwater	20-Jul-07	2	7.84	682	321	0.823
Candlesby	Groundwater	20-Jul-07	2	7.84	682	321	0.844
Candlesby	Groundwater	03-Dec-07	3	7.69	772	340	0.834
Candlesby	Groundwater	13-Mar-08	4	7.42	767	317	0.914
Catterick	Groundwater	01-May-07	1	7.43	647	260	0.154
Catterick	Groundwater	18-Jul-07	2	7.69	526	187	0.14
Catterick	Groundwater	28-Nov-07	3	7.40	670	268	0.159
Catterick	Groundwater	12-Mar-08	4	7.28	645	249	0.161
Great Wratting	Groundwater	30-Mar-07	1	7.28	1080	223	1.06
Great Wratting	Groundwater	20-Jul-07	2	7.62	929	354	0.984
Great Wratting	Groundwater	04-Dec-07	3	7.44	1120	364	0.962
Great Wratting	Groundwater	17-Mar-08	4	7.24	1110	352	0.915
Homesford	Sough	04-Apr-07	1	7.21	621	162	1.51
Homesford	Sough	17-Jul-07	2	7.40	583	189	1.51
Little Eaton	River	04-Apr-07	1	8.24	651	123	0.778
Little Eaton	River	17-Jul-07	2	7.00	233	47.5	0.303
Little Eaton	River	27-Nov-07	3	7.60	629	155	0.572
Little Eaton	River	11-Mar-08	4	8.29	629	169	0.624
Loxley Reservoir	Reservoir	01-May-07	1	7.70	216	6.8	<0.03
Loxley Reservoir	Reservoir	18-Jul-07	2	7.77	194	9.39	<0.06
Loxley Reservoir	Reservoir	28-Nov-07	3	7.59	201	119	<0.03
Loxley Reservoir	Reservoir	12-Mar-08	4	7.75	197	116	<0.03
Mynydd Llandegai	Reservoir	02-Apr-07	1	8.03	71.0	18.0	<0.03
Mynydd Llandegai	Reservoir	16-Jul-07	2	7.37	97.0	14.5	<0.03
Mynydd Llandegai	Reservoir	29-Nov-07	3	7.80	97.2		<0.03
Mynydd Llandegai	Reservoir	10-Mar-08	4	7.16	81.2		<0.03
Mythe	River	04-Apr-07	1	7.60	721	109	0.805
Mythe	River	16-Jul-07	2	7.15	506	85.3	0.655
Mythe	River	26-Nov-07	3	7.24	628	106	0.655
Mythe	River	10-Mar-08	4	7.22	582	124	0.613
Ogston Reservoir	Reservoir	04-Apr-07	1	7.56	446	59.7	0.410
Ogston Reservoir	Reservoir	17-Jul-07	2	7.76	370	61.6	0.362
Ogston Reservoir	Reservoir	27-Nov-07	3	7.66	481	109	0.937
Ogston Reservoir	Reservoir	11-Mar-08	4	7.26	440	104	0.120

Alkalinity measurements also indicate a large variability in chemical composition of the waters, ranging from soft water deriving from an upland Welsh reservoir being treated at Mynydd Llandegai (alkalinity values ≤ 18 mg/l as HCO_3_), through to hard Chalk groundwater being treated at Great Wratting (alkalinity values in the range 222–364 mg/l). Considerable variability was also observed in concentrations of a number of trace elements between sites, especially in the river water samples. However, no clear temporal trends are apparent from the available data.

**Fig. 2:**
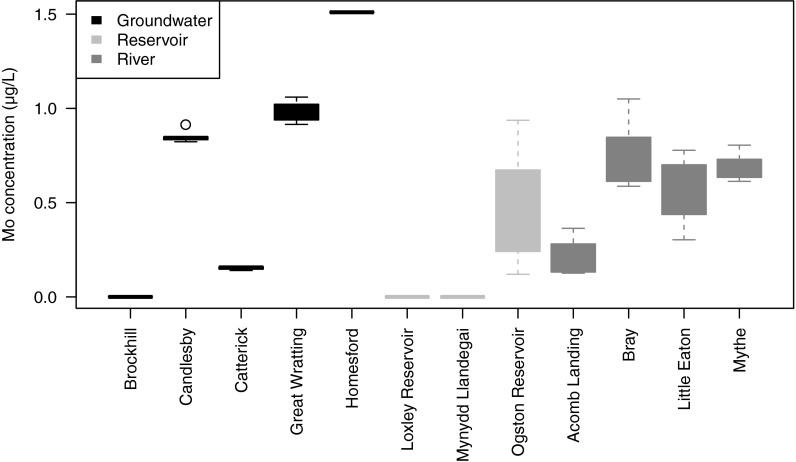
Box plots showing the variation in monitored Mo concentration from treated drinking water samples

Variations in Mo concentrations between sampling rounds at each of the sites are shown as box plots in Fig. 2. For Brockhill (groundwater), Loxley (reservoir), and Mynydd Llandegai (reservoir), all measured Mo concentrations were below the detection limit of 0.03 μg/l. Of the sources with detectable Mo, there appears to have been more temporal variability in concentrations in surface water sources than in groundwaters, consistent with variations induced by variable flow and dilution. Fig. 2 also suggests that overall median concentrations were slightly higher in groundwater sources compared to surface water sources.

The highest observed concentrations occurred in groundwater from Homesford, a supply abstracting from the mine sough. However, even in these cases, concentrations were only 1.5 μg/l. The sough is some 6 km long, and so, some attenuation of metals and dilution of mine drainage water is probable. All sources had concentrations well below the WHO health-based value for Mo of 70 μg/l.

Exploratory data analysis suggested that the concentrations were sufficiently normally distributed not to require transformation before carrying out statistical analysis to determine differences between source concentrations and between batches. We used a linear model (lm) fitted using the R routine lm (http://www.r-project.org/), applied separately to groundwater and surface water sources and restricted to sites with detectable Mo concentrations. The analysis showed significant differences between sources (*p* < 0.05), but no significant difference (*p* > 0.05) between samples within sites. There is therefore no evidence of a consistent seasonal effect from the data available.

### Domestic tap water survey

#### Molybdenum

**Fig. 3:**
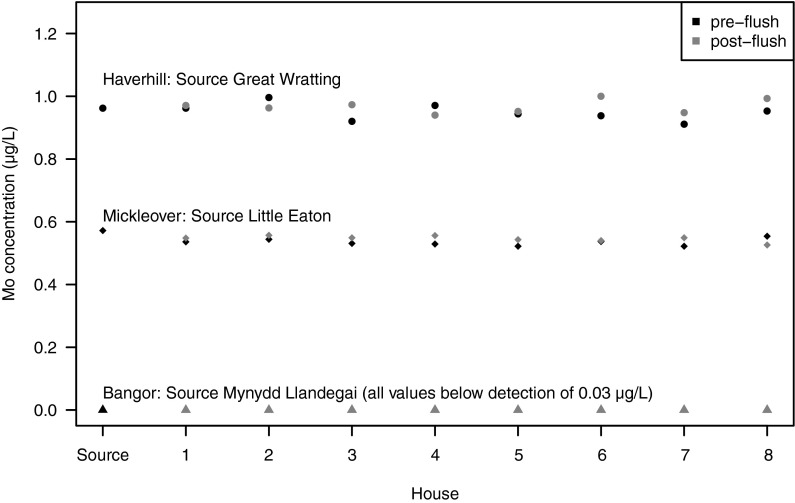
Molybdenum concentrations in tap water samples from three surveyed locations compared to their respective public supply sources [households 1–4 are ‘new’ properties (post-1990), while 5–8 are ‘older’ properties (pre-1960)]

Chemical data for selected elements from the tap water survey are given in Table 3. The relationship between public supply source and tap water Mo concentrations for the three surveyed areas is shown in Fig. 3. The uniformity in Mo concentrations in each source area is noteworthy, in comparison with concentration differences between sources. Model fitting showed no significant difference (*p* > 0.05) in Mo concentrations between houses (old and new), between pre-flush and post-flush samples, and between the tap water and source water samples. This suggests that tap water concentrations for these samples were largely unaffected by processes occurring within the water distribution pipework.

**Table 3:** Concentrations of selected trace elements in tap waters from the three towns

Locality	Mo (μg/l)	As (μg/l)	Cd (μg/l)	Co (μg/l)	Cr (μg/l)	Cu (μg/l)	Mn (μg/l)	Ni (μg/l)	Pb (μg/l)	Zn (μg/l)
Mickleover 1A-N	0.536	0.266	0.316	0.082	0.082	7.48	1.00	5.83	0.672	164
Mickleover 1B-N	0.548	0.291	0.281	0.093	0.126	1.04	1.75	2.31	1.86	60.2
Mickleover 2A-N	0.544	0.282	0.371	0.079	0.070	48.1	1.02	2.78	0.725	193
Mickleover 2B-N	0.557	0.257	0.310	0.076	0.197	3.91	0.815	2.26	0.645	64.3
Mickleover 3A-N	0.531	0.297	0.339	0.096	0.140	13.1	1.88	4.09	0.919	180
Mickleover 3B-N	0.549	0.30	0.290	0.086	0.168	1.38	1.40	2.38	1.00	62.6
Mickleover 4A-N	0.529	0.285	0.363	0.074	0.115	19.1	0.871	2.29	0.769	170
Mickleover 4B-N	0.556	0.278	0.332	0.076	0.126	1.70	0.809	2.18	0.674	67.4
Mickleover 5A-O	0.522	0.289	0.353	0.060	0.136	18.5	0.813	2.19	3.06	82.6
Mickleover 5B-O	0.543	0.292	0.352	0.067	0.164	1.62	0.726	1.88	2.67	67.8
Mickleover 6A-O	0.537	0.283	0.337	0.067	0.153	3.56	0.675	1.85	1.80	68.3
Mickleover 6B-O	0.540	0.295	0.330	0.066	0.119	4.93	0.676	1.83	1.67	65.4
Mickleover 7A-O	0.522	0.317	0.356	0.069	0.101	24.6	1.08	2.23	1.90	85.9
Mickleover 7B-O	0.549	0.285	0.312	0.075	0.165	2.20	1.08	2.20	1.20	63.8
Mickleover 8A-O	0.554	0.313	0.345	0.101	0.160	6.49	2.28	2.32	4.86	73.7
Mickleover 8B-O	0.526	0.313	0.279	0.095	0.156	1.34	2.09	2.19	3.06	59.1
Bangor 1A-N	<0.03	0.259	0.004	0.017	0.191	106	3.01	0.499	1.56	7.52
Bangor 1B-N	<0.03	0.242	0.003	0.015	0.235	7.14	2.75	0.262	0.061	1.78
Bangor 2A-N	<0.03	0.285	0.007	0.023	0.197	58.0	3.14	6.66	0.175	17.6
Bangor 2B-N	<0.03	0.247	0.004	0.016	0.238	3.13	2.88	0.501	<0.060	2.31
Bangor 3A-N	<0.03	0.253	0.004	0.015	0.167	33.4	2.23	0.697	0.512	5.67
Bangor 3B-N	<0.03	0.248	0.004	0.015	0.233	3.75	2.74	0.355	<0.060	2.59
Bangor 4A-N	<0.03	0.267	0.006	0.018	0.214	87.9	2.76	0.610	0.464	9.92
Bangor 4B-N	<0.03	0.239	0.007	0.020	0.204	54.4	3.88	0.627	0.364	18.9
Bangor 5A-O	<0.03	0.236	0.005	0.020	0.176	87.4	3.05	0.916	0.633	20.4
Bangor 5B-O	<0.03	0.254	<0.002	0.016	0.275	9.42	2.63	0.241	0.438	1.70
Bangor 6A-O	<0.03	0.238	0.003	0.014	0.167	36.9	2.18	0.511	3.29	13.3
Bangor 6B-O	<0.03	0.255	0.003	0.015	0.237	13.6	2.48	0.264	1.83	4.18
Bangor 7A-O	<0.03	0.235	0.006	0.018	0.169	94.9	2.46	1.53	199	3.28
Bangor 7B-O	<0.03	0.260	0.004	0.015	0.225	5.09	2.55	0.414	4.47	1.65
Bangor 8A-O	<0.03	0.242	0.005	0.017	0.156	11.3	3.07	0.970	0.113	4.85
Bangor 8B-O	<0.03	0.24	0.003	0.015	0.201	3.72	2.65	0.482	<0.060	2.67
Haverhill 1A-N	0.962	3.29	0.020	0.422	<0.040	76.3	0.25	15.85	0.364	99.9
Haverhill 1B-N	0.971	3.26	0.003	0.410	0.067	6.46	0.241	3.96	<0.060	10.8
Haverhill 2A-N	0.996	3.45	0.010	0.441	0.049	86.4	0.456	5.05	0.179	76.6
Haverhill 2B-N	0.963	3.33	<0.002	0.414	0.110	9.90	0.221	4.15	<0.060	11.8
Haverhill 3A-N	0.920	3.20	0.004	0.404	<0.040	79.9	0.253	4.21	0.218	32.3
Haverhill 3B-N	0.973	3.33	0.003	0.41	0.102	8.40	0.199	3.91	<0.060	14.6
Haverhill 4A-N	0.971	3.40	0.008	0.431	<0.040	34.7	0.263	4.32	0.255	38.7
Haverhill 4B-N	0.940	3.42	0.004	0.462	0.079	7.38	0.415	4.30	0.113	14.1
Haverhill 5A-O	0.944	3.03	0.041	0.405	<0.040	295	0.367	26.2	8.96	93.3
Haverhill 5B-O	0.952	3.36	0.003	0.391	0.142	21.4	0.165	5.19	3.63	9.51
Haverhill 6A-O	0.938	3.35	0.014	0.393	0.042	41.3	0.172	4.55	5.10	123
Haverhill 6B-O	1.00	3.30	<0.002	0.404	0.042	4.07	0.16	4.22	1.44	8.65
Haverhill 7A-O	0.911	3.19	0.010	0.368	0.075	141	0.131	5.05	1.90	44.7
Haverhill 7B-O	0.948	3.22	0.002	0.401	<0.040	4.64	0.139	4.39	1.55	5.56
Haverhill 8A-O	0.953	3.32	0.029	0.412	<0.040	48.5	0.148	6.64	11.2	147
Haverhill 8B-O	0.993	3.38	0.005	0.411	<0.040	3.36	0.167	4.41	2.29	9.60

*N* New house (post-1990), *O* old house (pre-1960), *A* Pre-flush sample (first morning draw), *B* post-flush sample, same tap

Of the areas surveyed, the greatest variation in Mo concentrations between houses occurred at Haverhill. However, the differences between houses or between tap water and source water were not significant (*p* > 0.05).

Data for Mo in tap waters from Mickleover also showed that concentrations did not differ significantly between houses. Tap water concentrations were lower than source concentration, and while this difference was statistically significant (*p* < 0.001), the magnitude of the difference was very small (0.03 μg/l) in relation to the concentrations measured. At Mickleover, source water and tap water were sampled on consecutive days. Since the Mickleover source is the River Derwent (Little Eaton), which had comparatively variable Mo concentration (Fig. 2), the difference in timing of sampling may be the cause of the difference between source and tap water concentrations. There is an indication that post-flush concentrations were significantly higher than pre-flush for this source (*p* = 0.04), although the differences were again very small (0.01 μg/l).

At Bangor, all analysed Mo concentrations in the tap water samples were below the detection limit of 0.03 μg/l and corresponded with the concentrations determined at the treatment works.

#### Other trace elements

Data for other trace elements determined at the same time as Mo showed some notable differences in chemistry between areas, consistent with the chemical compositions of their respective source waters. These data were log-transformed before analysis using the lm routine to determine differences between houses in each region and differences between pre-flush and post-flush water. Model performance was assessed by Akaike Information Criterion (AIC) minimisation and significance quoted for effects leading to a reduction in AIC between models.

One striking observation in the dataset was the difference in concentration of some trace elements between pre-flush and post-flush samples. Judged by AIC, pre-flush samples uniformly contained much higher concentrations of Ni, Cu, Zn, Pb, and Cd than post-flush samples. However, in almost all cases, pre-flush waters did not show concentrations above drinking water standards (Table 3). These elements are most likely derived from the metal pipework in the distribution system (Veschetti et al. 2010) and are therefore most concentrated in the water stored in the pipes overnight. One pre-flush sample had a concentration of Ni above the national drinking water limit for Ni of 20 μg/l (26 μg/l), and two had concentrations above the 2013 drinking water limit for Pb of 10 μg/l (199 and 11.2 μg/l). All post-flush samples had concentrations well below the respective limits.

Judged by AIC, at Haverhill and Mickleover, the concentrations of Pb in samples from old houses (pre-1960) were significantly higher (*p* < 0.05) than for new (post-1990) houses (see also Table 3). In Bangor, some individual older houses showed higher Pb concentrations than new houses, while others did not. These differences are likely due to the presence of lead pipes (and/or solder) in the domestic plumbing systems. Note that new and old houses were sampled from within a single random street for each age class. They were not a random sample of old and new houses from each locality. On the basis of statistical analysis, it is therefore not possible to make inferences about tap water samples taken in streets not surveyed. Tentative extrapolation may be made on the basis of process understanding.

There was also a tendency (*p* < 0.05 for Bangor; *p* < 0.1 for Haverhill and Mickleover) for post-flush samples to have slightly higher concentrations of Cr than pre-flush samples, although as with Mo, the magnitude of the increases was small. All samples had concentrations at least two orders of magnitude less than the drinking water limit for Cr of 50 μg/l.

The observations of increased relative concentrations of Ni, Cu, Zn, Pb, and Cd in pre-flush compared to post-flush tap waters and in Pb in old compared to new properties reinforce the contrast with Mo, which showed comparatively little variation that could be attributed to contamination from distribution pipe networks and domestic plumbing.

## Discussion

Molybdenum concentrations in water are impacted by a combination of available sources and ambient water chemistry, particularly, pH and redox conditions. In oxic water at pH > 5, Mo occurs principally as the molybdate oxyanion (MoO_4_^2-^), which is known to adsorb readily to iron oxides (Dzombak and Morel 1990; Kaback and Runnells 1980; Morrison and Spangler 1992) and aluminium oxides (Goldberg et al. 1996) under near-neutral and acidic pH conditions. Molybdate also adsorbs to manganese oxides and some clays under acidic conditions. Mobility of the oxyanion is favoured under more alkaline pH conditions (Dzombak and Morel 1990; Kaback and Runnells 1980; Morrison and Spangler 1992). Under reducing conditions in soils and aquifers, mobilisation of Mo can occur in response to reductive dissolution of Fe and Mn oxides (Bennett and Dudas 2003; Schlieker et al. 2001). However, under strongly reducing conditions in the presence of sulphide, immobilisation has been attributed to the reduction of Mo(VI) molybdate to Mo(IV) and resultant co-precipitation with FeS or FeS_2_ (Helz et al. 2004), or potentially precipitation as MoS_2_ (e.g. Amrhein et al. 1993). However, the kinetics of the latter reaction are noted to be slow (Bostick et al. 2003; Erickson and Helz 2000), and the mineral is rarely observed in nature. Redox zonation can therefore result in spatial variation in dissolved Mo concentrations, which has been observed in column experiments (Schlieker et al. 2001) and aquifers (Smedley and Edmunds 2002).

Assuming the Mo data collected in the surveys are representative of British source waters and treated tap waters as a whole, concentrations of Mo are at least an order of magnitude below the health-based value of 70 μg/l proposed for drinking water by WHO (2011b). The finding suggests that Mo in drinking water in England and Wales is unlikely to pose a problem for supply or human health. This is consistent with observations for typical surface water and groundwater in the UK (Smedley et al. 2014). It is also consistent with observations from many source waters and tap waters elsewhere (e.g. Dinelli et al. 2012; Frengstad et al. 2000; Van Geen et al. 2007), although it does not preclude high concentrations occurring sporadically in waters impacted by sulphide mineral oxidation (Leybourne and Cameron 2008) or industrial contamination (Leung and Jiao 2006). Relatively high Mo concentrations have been found in groundwater under oxic, alkaline conditions in some volcanic terrains, albeit such occurrences rarely appearing to exceed 70 μg/l. Smedley and Nicolli (2014) reported concentrations of Mo up to 990 μg/l (range 2.7–990 μg/l, median 61.5 μg/l, *n* = 114) under oxic and alkaline conditions in groundwater impacted by rhyolitic ash deposits from the Chaco-Pampean Plain of Argentina. Here, 40 % did exceed 70 μg/l. Reimann et al. (2003) reported concentrations in the range < 0.002–78.3 μg/l (median 2.93 μg/l, *n* = 138) for groundwater of mostly oxic, alkaline character from the Ethiopian Rift Valley. Just 2 % exceeded 70 μg/l. Groundwater from the alluvial Willcox Basin aquifer of Arizona, USA, has a reported range in Mo of 1.0–11.1 μg/l, the highest concentrations occurring under oxic, alkaline conditions and concluded to derive from weathered felsic volcanic material in the sediments (Vinson et al. 2011). The concentrations were high by world standards, but none in this case exceeded or approached the WHO health-based value.

The observations for the British drinking waters lend further support to the WHO (2011a, b) decision to remove Mo from the list of formal guideline values on the basis that such concentrations are rarely encountered in drinking water. This removes the obligation to introduce Mo as a regulated trace element in national drinking water legislation, with consequent routine monitoring requirements and costs. In specific cases where the risk of exceedance of the 70 μg/l health-based recommendation for Mo is increased, separate provisions for surveillance and monitoring at a local or national level are needed.

Previous studies of drinking water chemistry have also reported high concentrations of Fe, Cu, Zn, Cr, Ni, and Pb as a result of contamination from plumbing infrastructure. Several studies have shown high concentrations in first draw samples of tap water compared with fully flushed samples (Andersen et al. 1983; Gulson et al. 1997; Rajaratnam et al. 2002). The Veschetti et al. (2010) study of Italian tap water also reported problems with Fe, Ni, and Pb. These were attributed to corrosion, contamination from domestic taps and from lead pipes in old buildings, respectively. The Veschetti et al. (2010) investigation did not find significant difference in concentrations of these elements between random daytime non-flushed and flushed samples, although in this case, they were not first morning-draw samples. Lack of variation with flushing was also reported for Pb by Baron (2001).

Since the collection of tap water samples in our study, the 2013 European legislation stipulating a reduction in the Pb limit to 10 μg/l at consumers’ taps has resulted in a large expansion of plumbosolvency measures in England and Wales, principally via orthophosphate dosing. This is reported to be highly effective in reducing the Pb concentration in supplied tap water (Hayes and Hoekstra 2010), and so, actions to mitigate the exceedance for Pb have been taken. More than 95 % of public drinking water supplies in England and Wales are now phosphate-dosed to control Pb concentrations (CIWEM 2011).

## Conclusions

The distribution of Mo in drinking waters from England and Wales indicates that mobility is limited and that concentrations are usually low in relation to the WHO health-based criteria. All sources analysed had concentrations < 2 μg/l. These results are consistent with the findings for raw source waters in Britain (Smedley et al. 2014). The data for Mo from the survey of 12 public supply sources and tap waters from three of the supplied areas indicated a clear variability in concentrations between sites and sources. The highest concentrations were observed in groundwater abstracted from a mine drainage tunnel (sough), consistent with observed high values in metal-mineralised areas from source water data. However, even at this site, the concentrations were only 1.5 μg/l. Other sites did not show evidence of anomalously high concentrations that had been observed in surface waters or groundwaters (Table 1) from the corresponding sources. Lowest concentrations were found in treated water from upland reservoir sites, here probably related to limited contact with bedrock materials, and with the most acidic groundwater represented (Brockhill, ca. pH 6.6) in which Mo concentrations are likely limited by binding of molybdate to metal oxides.

Concentrations of Mo were remarkably consistent within sites. Despite evidence from surface-water data for concentrations of a number of solutes varying with stream discharge (Neal et al. 2003; Neal and Robson 2000; Wilkinson et al. 1997), monitoring at each treatment works revealed no discernible temporal trends.

Of the tap water samples taken from three towns, those supplied from the reservoir source had Mo concentrations universally below detection limit (0.03 μg/l), consistent with concentrations in the public supply source water. Tap waters derived from the Chalk groundwater were not significantly different from their source waters (*p* > 0.05). Tap waters derived from the river water had lower Mo concentrations than their source waters. The differences were statistically significant (*p* < 0.001), but their absolute magnitude was small. These results suggest that inputs of Mo to the drinking water from the pipe distribution and storage system were negligible.

In the cases where there was an indication of slightly higher Mo and Cr concentrations in post-flush tap water samples compared to pre-flush samples, the cause is unclear. It is possible that some adsorption of these metals, both occurring as oxyanions in oxic neutral pH waters, has occurred onto pipework, solder, or any encrusted minerals in the plumbing system. This might be expected to affect water stored overnight to a greater extent than flushed water, although the conclusion remains speculative without further data. Nonetheless, the increases in post-flush samples compared to first morning-draw samples were of small magnitude and not of practical significance.

Significant differences were found between pre-flush and post-flush samples in concentrations of the trace metals Pb, Ni, Cu, Zn, and Cd. Highest concentrations were present in pre-flush waters, and for Pb, higher concentrations were usually found in pre-flush waters from older properties. The drinking water limits for Pb (2013 limit: 10 μg/l) or Ni (20 μg/l) were exceeded in three pre-flush samples, although all post-flush samples had concentrations below the respective limits.

The probability of a drinking water source in England and Wales exceeding 70 μg/l Mo could not be computed from the data produced in this study. Such an event would be so far from the measured values as to be outside the range of meaningful extrapolation. If the Mo data collected in the surveys are representative of British source waters and tap waters as a whole, then concentrations of Mo are at least an order of magnitude below the WHO health-based value for drinking water of 70 μg/l and therefore unlikely to pose a problem for water supply or health. This is consistent with the WHO (2011a, b) decision to remove Mo from the list of formal guideline values on the basis that such concentrations are rarely encountered in drinking water.
